# Retraction: An improved DBSCAN algorithm based on cell-like P systems with promoters and inhibitors

**DOI:** 10.1371/journal.pone.0345270

**Published:** 2026-03-19

**Authors:** The PLOS One Editors

The *PLOS One* Editors retract this article [1] due to concerns about compromised peer review. We regret that the issues were not addressed prior to the article’s publication.

All authors either did not respond directly or could not be reached.
